# Humoral Autoimmune Responses to Insulin-Like Growth Factor II mRNA-Binding Proteins IMP1 and p62/IMP2 in Ovarian Cancer

**DOI:** 10.1155/2014/326593

**Published:** 2014-04-27

**Authors:** Xinxin Liu, Hua Ye, Liuxia Li, Wenjie Li, Yi Zhang, Jian-Ying Zhang

**Affiliations:** ^1^Center for Tumor Biotherapy, The First Affiliated Hospital & College of Public Health, Zhengzhou University, Zhengzhou, Henan 450052, China; ^2^Department of Biological Sciences & Border Biomedical Research Center, The University of Texas at El Paso, El Paso, TX 79968, USA

## Abstract

Ovarian cancer is one of the leading causes of cancer-related deaths among women. There is an urgent need of better approaches for the identification of appropriate biomarkers in the early detection of ovarian cancer. The aim of this study was to elucidate the significance of autoantibodies against insulin-like growth factor II mRNA-binding proteins (IMPs) in patients with ovarian cancer. In this study, autoantibody responses to two members (IMP1 and p62/IMP2) of IMPs were evaluated by enzyme-linked immunosorbent assay (ELISA), western blotting, and indirect immunofluorescence assay in sera from patients with ovarian cancer and normal human individuals. The results have demonstrated that both IMP1 and p62/IMP2 can induce relatively higher frequency of autoantibody responses in patients with ovarian cancer (26.5% and 29.4%) compared to normal individuals (*P* < 0.01). Our preliminary data suggest that IMP1 and p62/IMP2 can stimulate autoimmune responses in ovarian cancer, and anti-IMP1 and anti-p62/IMP2 autoantibodies could be used as potential biomarkers in immunodiagnosis of ovarian cancer.

## 1. Introduction

Autoantibodies are well known for their pathological role in autoimmune diseases, such as rheumatoid arthritis or systematic lupus erythematosus [1]. Cancer onset and progression produce mutated or aberrantly expressed proteins which are able to act as antigens and evoke an immune response, a process which results in the production of autoantibodies. These autoantibodies are able to be detected several months or several years before the clinical diagnosis of cancer [2–4], and therefore tumor-associated antigens (TAAs) and their corresponding autoantibodies could be used as biomarkers for the early diagnosis and prognosis of cancer [5–8]. Autoantibodies represent an immunological fingerprint in the pathological progression of cancer, and tumor-induced antibodies may be able to provide a unique insight into host-tumor interactions and the dynamic nature of carcinogenesis [6, 9–11].

Ovarian cancer is currently the leading cause of mortality among gynecological malignant tumors, with epithelial ovarian cancer being the most common, accounting for >85% of all clinical cases [12]. The majority of ovarian cancers are diagnosed at an advanced stage, mostly due to a lack of effective screening strategies and difficulties in obtaining an efficient diagnosis [13]. It has generally been assumed that if ovarian cancer could be diagnosed at an early stage, this would result in a significant improvement in survival [14]. It is well accepted that early diagnosis can improve survival; thus, there is a great need and anticipation to identify novel biomarkers for ovarian cancer diagnostics at the earliest stage.

The insulin-like growth factor II mRNA-binding proteins 1 and 2 (IMP1, p62/IMP2) belong to a conserved family of RNA-binding proteins. Several studies have shown that these proteins act in various important aspects of cell function, such as cell polarization, migration, morphology, metabolism, proliferation, and differentiation [15]. IMPs are primarily expressed during early embryogenesis and at midgestation in the mouse [16]. Importantly, IMPs are frequently overexpressed in various cancers and are considered to be oncofetal proteins [17–19]. Whether autoantibodies to IMP1 and p62/IMP2 can be used as the biomarkers for the diagnosis and prediction of ovarian cancer, and the mechanism of immune responses to IMP1 and p62/IMP2 in ovarian cancer remains to be investigated and evaluated. In the present study, we determined the frequency of antibodies to IMP1 and p62/IMP2 in ovarian cancer patients and evaluated the usefulness of anti-IMP1 and anti-p62/IMP2 antibodies as biomarkers for the diagnosis of ovarian cancer.

## 2. Materials and Methods

### 2.1. Patients and Samples

In the current study, total 34 sera from patients with ovarian cancer and 89 sera from normal individuals were obtained from the sera bank in The Cancer Autoimmunity Research Laboratory at The University of Texas, El Paso (UTEP). These sera were originally provided by our clinical collaborators. All ovarian cancer sera were collected at the initial time of cancer diagnosis, prior to patients being treated with chemotherapy or radiotherapy. Normal human sera were assembled during annual health examinations from adults with no obvious evidence of malignancy. Due to regulations concerning studies on human subjects, patients' name and identification number were not disclosed to investigators, and some clinical information for sera used in the study was not available. This study was approved by the Institutional Review Board of UTEP and Collaborating Institutions.

### 2.2. Enzyme-Linked Immunosorbent Assay (ELISA)

Serum IgG antibodies against IMP1 and p62/IMP2 were measured by ELISA as previously described [20]. In brief, the 96-well microtiter plates were coated overnight (at least for 24 h) at 4°C with 0.5 *μ*g/mL IMP1 and 2 *μ*g/mL p62/IMP2 diluted in phosphate-buffered saline (PBS), respectively. Plates were blocked with gelatin postcoating solution for 2 h at room temperature. The antigen-coated wells were incubated with human sera diluted at 1 : 200 with serum diluent at room temperature for 2 h. The goat anti-human IgG-HRP (Invitrogen, NY) and the substrate 2,2′-azino-bis-3-ethylbenzo-thiazoline-6-sulfonic acid (ABTS, Invitrogen) were used as detecting reagent. The average optical density (OD) value at a wavelength of 405 nm was applied for data analysis. The cutoff value designating positive reaction was the mean OD of 89 normal human sera (NHS) plus 3 standard deviations (SD).

### 2.3. Western Blotting

Purified recombinant IMP1 and p62/IMP2 proteins were electrophoresed on 10% SDS-PAGE and transferred onto a nitrocellulose membrane paper. After blocking with PBS containing 5% nonfat dry milk and 0.05% Tween-20 (PBST) for 1 h at room temperature, the nitrocellulose papers were incubated for 60 min at room temperature with a 1 : 200 dilution of serum and 1 : 500 dilution of monoclonal anti-IMP1 and monoclonal anti-p62/IMP2 antibodies. HRP-conjugated goat anti-human IgG and HRP-conjugated goat anti-rabbit IgG were applied as secondary antibody at a 1 : 1,000 dilution. Immunoreactive bands were detected using the ECL kit (Thermo Scientific, MA) according to the manufacturer's instructions.

### 2.4. Indirect Immunofluorescence (IIF) Assay

Commercially available HEp-2 cell slides (MBL International Corporation, MA) were used in IIF for identification of autoantibodies in cancer sera. Sera with 1 : 80 dilution and monoclonal anti-IMP1 and anti-p62/IMP2 antibodies with 1 : 20 dilution were incubated for 1 h at room temperature. FITC-conjugated goat anti-human IgG (1 : 200 dilution), anti-mouse IgG Fab2 Alexa Fluor (1 : 50 dilution), and goat anti-rabbit IgG FITC (1 : 50 dilution) were used as secondary antibodies, respectively. Immunofluorescence images were acquired with a laser scanning confocal microscope (LSM 700; Zeiss, New York, NY) using a 20x objective and processed with ZEN 2009 software (Zeiss, CA).

### 2.5. Absorption of Antibodies with Recombinant Protein

The diluted human sera (1 : 80) were incubated with recombinant protein (final concentration of recombinant protein was 0.03 *μ*g/*μ*L) overnight at 4°C and then centrifuged at 10,000 ×g for 15 min. The supernatant was used for immunofluorescence assay.

### 2.6. Statistical Analysis

Statistical analysis was performed using SPSS 13.0. Data were analyzed with Chi-square test and represented as the mean plus 3 standard derivation (SD) from ELISA. The results were considered to indicate a statistically significant difference when *P* value was less than 0.01.

## 3. Results

Frequency and titer of anti-IMP1 and anti-p62/IMP2 autoantibodies in human ovarian cancer sera.

**Figure 1:**
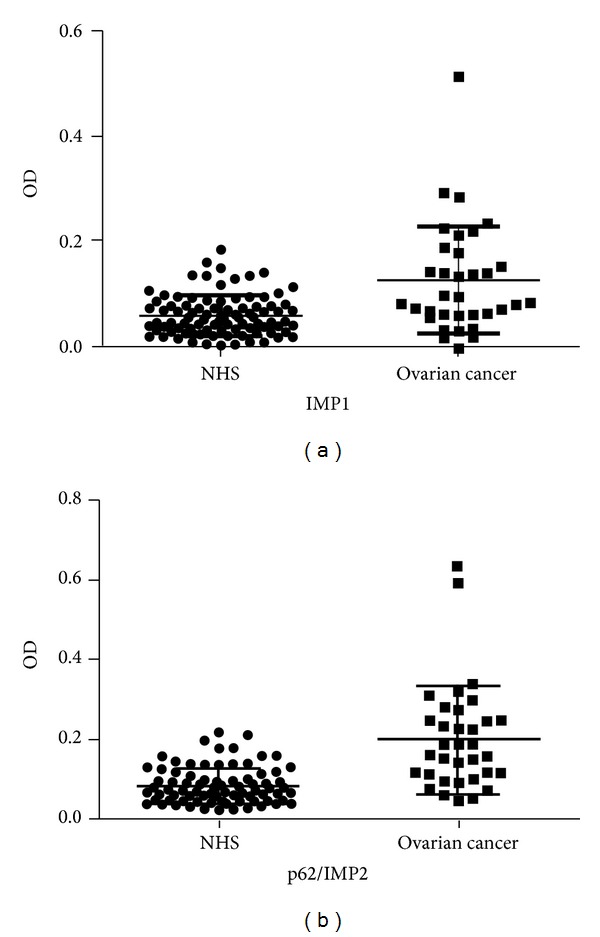
Titer of autoantibodies against IMP1 and p62/IMP2 in human sera by ELISA. The range of antibody titers to IMP1 and p62/IMP2 was expressed as optical density (OD) obtained from ELISA. The mean + 3SD of NHS is shown in relationship to all serum samples. Titer of anti-IMP1 and anti-p62/IMP2 in ovarian cancer is much higher than that in NHS (*P* < 0.01).

**Figure 2:**
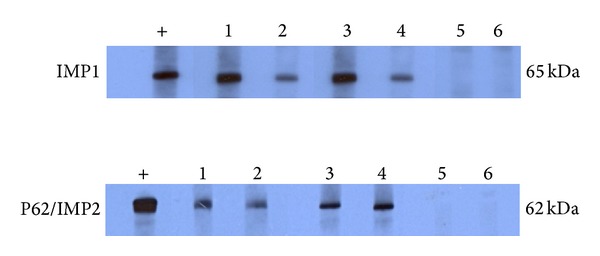
Western blotting analysis showing representative ovarian cancer sera recognizing IMP1 and p62/IMP2 recombinant proteins. The monoclonal anti-IMP1 and anti-p62/IMP2 antibodies were used as positive controls; lanes 1–4, four representative ovarian cancer sera that were positive in ELISA test and also have strong reactivity with IMP1 and p62/IMP2 recombinant proteins in Western blotting analysis; lanes 5 and 6, normal human sera that were used as negative control.

Serum levels of anti-IMP1 and anti-p62/IMP2 autoantibodies were determined by ELISA as described in the section of Materials and Methods. In total, 34 sera from patients with ovarian cancer and 89 sera from normal human individuals were used in this study. As shown in Table 1, the prevalence of autoantibody against IMP1 was 26.5% (9/34) in ovarian cancer, which was significantly higher than that in NHS (1.1%, 1/89) (*P* < 0.01). Titer of anti-IMP1 antibody in human sera was shown in Figure 1. The average titer of autoantibody against IMP1 in ovarian cancer sera was higher than that in NHS (*P* < 0.01). As demonstrated in Table 1, the frequency of autoantibody to p62/IMP2 was 29.4% (10/34), which was significantly higher than that in NHS (1.1%, 1/89). Titer of anti-p62/IMP2 antibody in human sera was shown in Figure 1. The average titer of autoantibody against anti-p62/IMP2 in ovarian cancer sera was higher than that in NHS (*P* < 0.01). The ELISA results were also confirmed by western blot analysis.  Figure 2 showed that representative ovarian cancer sera with positive reaction to IMP1 and p62/IMP2 in ELISA also have strong reactivity in western blotting compared to normal sera.

### 3.1. Immunofluorescence Staining Pattern of IMP1 and p62/IMP2 in HEp-2 Cells

**Figure 3:**
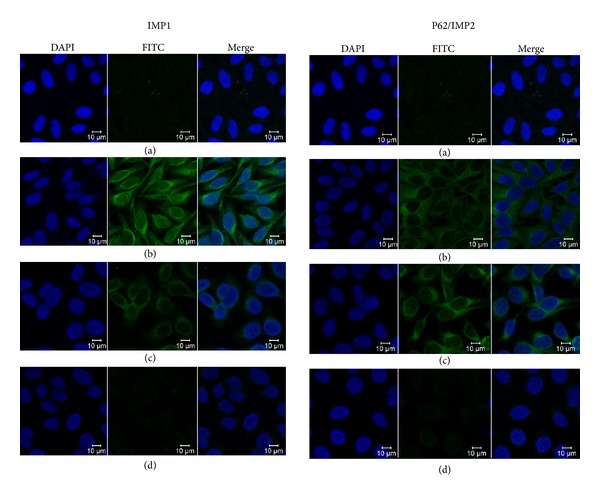
Representative immunofluorescence staining pattern of anti-IMP1 and anti-p62/IMP2 positive ovarian cancer sera. (a) A normal human serum (NHS) was used as negative control; (b) monoclonal anti-IMP1 or anti-p62/IMP2 antibody that demonstrated a cytoplasmic immunofluorescence staining pattern was used as positive control; (c) representative anti-IMP1 or anti-p62/IMP2 positive ovarian cancer sera demonstrated an intense cytoplasmic immunofluorescence staining pattern; (d) the same ovarian cancer serum that was used in panel (c) was postabsorbed with recombinant IMP1 or p62/IMP2 protein. The fluorescent cytoplasmic signal was remarkably decreased.

To further confirm the reactivity of autoantibodies against members of IMPs in ovarian cancer sera and the intracellular localization of IMPs, HEp-2 cell slides were used in indirect immunofluorescence assay to detect ovarian cancer sera with anti-IMPs positive in ELISA. As shown in Figure 3, a representative ovarian cancer serum with anti-IMP1 antibody positive in ELISA had an intense cytoplasmic staining pattern, which was similar to the staining pattern shown by monoclonal anti-IMP1 antibody which is mainly located at the cytoplasm. The fluorescent cytoplasmic staining was significantly reduced when the same ovarian cancer serum was preabsorbed with recombinant IMP1 protein. As demonstrated in Figure 3, a representative anti-p62/IMP2 positive ovarian cancer serum had the cytoplasmic pattern, and the staining pattern with monoclonal anti-p62/IMP2 antibody is also located at the cytoplasm. The same ovarian cancer serum was preabsorbed with recombinant p62/IMP2 protein, and then the fluorescent signal in the cytoplasm was significantly decreased.

## 4. Discussion and Conclusion

The IMPs family (IMP1, p62/IMP2, and koc/IMP3) binds and regulates translation of insulin-like growth factor II mRNA. Members of this protein family are oncofetal proteins [21, 22], which have been implicated in RNA localization, stability, and translation which are essential for normal embryonic growth and development. The expression of these proteins disappears from all tissues soon after birth but frequently has appeared again during the process of malignant transformation. The overexpression of these proteins has been detected in many types of tumors [23–25], and it has been hypothesized that these proteins can mediate cell motility and invasion and might be closely related to cancer.

IMPs are predominately localized in cytoplasm, usually with a granular appearance. A nuclear role of IMPs remains controversial, although there was evidence that IMPs were associated with their target mRNAs at their site of transcription [26–28]. In agreement, IMPs were observed in the nucleus of spermatogenic cells and were suggested to comprise nuclear export signals [29]. In the cytoplasm, IMPs form distinct ribonucleoprotein (RNP) granules which are enriched in the perinuclear region but are also observed in neuritis of developing neurons supporting a role of IMPs in promoting mRNA localization [30, 31]. In consistence with our results, both IMP1 and p62/IMP2 proteins were expressed at a higher extent in cytoplasmic fractions. Therefore, the cytoplasmic IMP1 and p62/IMP2 proteins may have the functional role in ovarian carcinogenesis.

IMP1, which is almost identical to mouse coding region determinant-binding protein (CRD-BP) [32] and closely related to chicken zip-code binding protein 1(ZBP1), binds directly to and stabilizes oncogenic c-myc and regulates its posttranscriptional expression and translation [33, 34]. In addition to the determination of the localization and translation of *β*-actin mRNA, orthologs of IMP1 were also reported to regulate the translation of insulin-like growth factor II mRNA [35] and the stabilization of c-myc [33, 36, 37], *β*-actin [37], and *β*-TrCP1 mRNAs [37, 38]. A study has indicated that CRD-BP, the ortholog of IMP1, promotes cell proliferation by suppressing apoptosis [39]. Furthermore, CRD-BP positively influenced the ability of metastatic melanoma cells to proliferate and invade in response to hypoxia [40]. In contrast to its prooncogenic properties, a study has shown that the loss of CRD-BP induces leukemia cell proliferation [35] and repression of IMP1 expression leading to the increased proliferation and migration of metastatic breast cancer cells [41]. Studies from different groups all suggested that IMP1 might play an essential role in tumor progression. In the present study, the prevalence of autoantibody against IMP1 was 26.5% (9/34) in ovarian cancer, which was significantly higher than that in normal individuals.

Moreover, P62/IMP2, a cancer-associated antigen, was isolated by immunoscreening a cDNA expression library with autoantibodies from patients with hepatocellular carcinoma (HCC) [23]. It showed a significant homology with members from a family of mRNA binding proteins [24, 33, 34, 42, 43], containing an RNA recognition motif [44, 45] and four hnRNP K homology (KH) domains [46, 47]. Antibodies to p62/IMP2 were found in 21% of patients with HCC but not in the precursor conditions such as chronic hepatitis and liver cirrhosis [23]. Immunohistochemical analysis of HCC tissues showed that, in 33% of patients, cytoplasmic p62/IMP2 staining was significant in all malignant cells in cancer nodules, as in fetal livers, but it was undetectable in adjacent nonmalignant cells and normal adult livers. This might indicate that p62/IMP2 is associated with hyperproliferating cells [48]. In this study, the frequency of autoantibody to p62/IMP2 was 29.4% (10/34), which was significantly higher than that in sera with normal individuals.

Antitumor antibodies have also been detected in ovarian cancer [49]. However, relatively few antigens associated with antitumor antibodies in ovarian cancer have been identified compared with those reported for other cancers [50]. CA125 is a serum marker which has been approved to monitor ovarian cancer prognosis. Detection of abnormally elevated CA125 in plasma is correlated with tumor diameter; only 21% of patients has microscopic disease, but >70% with a tumor diameter of 1-2 cm have elevated values [51]. CA125 test alone lacks the specificity necessary for use as a population screening for early stage ovarian cancer. In addition, autoantibodies directed against the epithelial cell adhesion molecule (Ep-CAM), IL-8, type-1 angiotensin II receptor, and MUC1 could increase the sensitivity and specificity of the CA125 biomarker for ovarian cancer detection [52–55]. Combination of these cancer-related autoantibodies resulted in increased diagnostic power of the assay suggesting that circulating antibodies could potentially be valuable diagnostic markers. Further research is underway to analyze the role of multiple circulating antibodies for early detection and prognosis of ovarian cancer.

The immunogenic nature of ovarian cancer is a promising property of this cancer that can be exploited for the identification of a large number of tumor antigens involved in the pathogenesis of ovarian cancer. This tumor immunogenicity leads to the generation of large diversity of antibody repertoire directed against autologous tumor-related antigens. The detection of serum antibody responses to tumor antigens may provide more reliable serum biomarkers for cancer diagnosis because serum antibodies are more stable compared to serum antigens. Circulating serum antigens are more reliable and have the shorter half-life. For example, the reported half-life of CEA, CA19-9, and AFP was approximately 1.5 days, 0.5 days, and 1 day in patients after removal of intrathoracic malignancies [56], and the half-life of S100B protein in melanoma patients was reported to be 30 minutes [57]. In contrast, antibodies are more abundant than antigens, especially at low tumor burdens of early stage of cancers, and their role as reporters of early or incipient carcinogenesis has been well documented. Hafner et al. reported that anti-p53 autoantibody may be more sensitive than CA-125 in monitoring microscopic and macroscopic residual disease after primary therapy for epithelial ovarian cancer [58]. Therefore, a panel of TAA candidates for cancer immunotherapy should be selected in a way that activation of immune responses against those TAAs will have favorable clinical outcomes.

In summary, our data show that certain patients with ovarian cancer have preferential immune responses to IMP1 and p62/IMP2 in sera. Therefore, IMP1 and p62/IMP2 could become a target of therapeutic strategies in the malignant ovarian cancer. Further studies should be directed at selecting other tumor-specific autoantibodies and be attempted to design a unique anti-TAAs autoantibody panel for different types of cancer and to determine whether a miniarray of multiple anti-TAAs autoantibodies would be a useful approach for early detection and diagnosis of certain type of cancer.

## Figures and Tables

**Table 1:** Frequency of autoantibodies against IMP1 and p62/IMP2 in human sera by ELISA.

Autoantibodies	Number (%) of autoantibodies	
	Ovarian cancer (34)	NHS (89)
IMP1	9 (26.5)*	1 (1.1)
P62/IMP2	10 (29.4)*	1 (1.1)

Cutoff value: mean + 3SD of NHS; *P* value relative to NHS; *P < 0.01.
